# Lived Experiences of Mothers Raising Children with Autism in Chitwan District, Nepal

**DOI:** 10.1155/2021/6614490

**Published:** 2021-11-06

**Authors:** Sabitri Acharya, Kalpana Sharma

**Affiliations:** Chitwan Medical College, Affilated to Tribhuwan University, Chitwan, Bharatpur, Nepal

## Abstract

**Background:**

Autism is a neurodevelopmental problem that is increasing at an alarming rate worldwide. Rearing and caring for children with autism depends upon the perception of mothers and various factors associated with it. There is a gap in the literature regarding the detailed accounts of mother's experiences regarding autism in Nepal. Hence, this study was undertaken to explore lived experiences of mothers raising children with autism.

**Materials and Methods:**

Qualitative phenomenological study design was used and nine mothers with autistic children were selected using purposive sampling technique. Data were collected using in-depth interview guidelines and analyzed using Colaizzi's steps.

**Results:**

Findings of the study revealed that mothers raising children with autism encountered numerous problems in their life. They felt physically exhausted due to the continuous supervision of their child. Emotional problems such as denial, upset/sadness, and worry were also common among them. In addition, all mothers faced social problems such as social blame, social isolation, and ignorance from their relatives and society due to the atypical behavior of their child. Furthermore, the economic problem was also acute among mothers due to job loss, costly medical treatment, and therapies. So, to deal with the stressors they faced, mothers adopted various coping strategies such as respite care, problem-focused strategies, religious coping, and positive coping in their everyday life.

**Conclusion:**

In conclusion, to the authors' knowledge, this is the first study documenting the experiences of Nepalese mothers having autistic children. Hence, health care professionals need to pay more attention to address the problems of mothers while treating their autistic children. The Government of Nepal also needs to formulate a policy for the rehabilitation of autistic children in society.

## 1. Introduction

Autism is a pervasive neurodevelopmental disorder characterized by impairments in social communication and restricted, repetitive patterns of behavior, interests, or activities [1]. The Centers for Disease Control and Prevention biennial data from the Autism and Developmental Disabilities Monitoring Network (ADDM) on the prevalence of autism in the US showed 1 in 54 children have autism spectrum disorder (ASD), representing a 10% increase from previous estimates [2, 3]. Based on the tracking in 8-year-old children, within 11 communities in U.S, about 1.85%, or 1 in 54, was identified with ASD in 2016. Likewise, the reported prevalence of autism in South Asia ranged from 0.09% in India to 1.07% in Sri Lanka, which indicates that 1 in 93 children has autism in South Asian Region. This alarmingly high prevalence (3%) was reported in Dhaka city. Study sample sizes ranged from 374 in Sri Lanka to 18,480 in India, aged between 1 and 30 years. There are no studies that reported the prevalence of ASD in Pakistan, Bhutan, Maldives, and Afghanistan [4]. Similarly, there are no reliable estimates of autism in Nepal; however, Autism Care Society Nepal estimated that there are about 2,50,000–3,00,000 persons with Autism (PWAs) in Nepal [5].

Children with autism are enormously different from others. The core features, associated symptoms, and behavior problems of autism causes significant negative impact on families and parental well-being. Problems in communication and social skills, and behaviors that they experience, cause their parents to have various challenges. This leads to a life of chronic sadness and depression for parents, leading to negative attitudes and self-accusation rather than a positive approach to life [6]. Research has also illustrated the immense challenges to the individuals with autism as well as in their families and the impact on the parental well-being, which included emotional stress; ongoing financial burden of expensive treatments and therapies; significant strain on family relationships; changes in family roles, structure, and activities; and feeling of guilt and blame regarding diagnosis and social stigma [7].

The experiences of mothers vary from country to country. A study conducted in Australia showed that mothers of children with autism experience poorer health and well-being compared to mothers of children with other disabilities [8]. Mothers in the` United Kingdom experienced autism as mysterious and complex that cause self-injury, harm to others and damaged homes, unrelenting stress, and isolation from friends, school, the public [9]. Likewise, Canadian mothers presented poignant narratives about living with their autistic children. The study conducted in Korea showed that the autistic mothers face difficulty in accepting limits imposed by the disability's discouragement and suffering [10].

Parenting children with autism is more stressful and challenging than parenting children with typical development. Mother of a child diagnosed with autism often faces substantial challenges due to the child's disability that can be manifested in multiple ways and cause tension for the mother as well as the entire family [11]. The common stressors include a sense of loss and depression, decreased opportunities for familial time and outings, changes in relationships resulting in loss of social support, and personal sacrifices. The period immediately after child's diagnosis is unacceptable for mothers and they denied the diagnosis. They felt confused, sad, guilt, and even may experience depression. In addition, mothers also feel a deep sense of loss as they are forced to abandon their dreams and expectations for the child's future. Moreover, concerning the socially unacceptable behavior of the child, mothers restrict themselves from enjoying many enjoyable activities, thus creating isolation, declining social support, and likely perpetuating stress [12]. Likewise, many parents are forced to leave a career to provide full-time care to the child, manage the treatment, and to pursue quality resources [13].

Limited research has explored the experience of mothers caring for their autistic children in low-income countries and non-western countries compared to high-income countries. For instance, a qualitative study conducted in Malaysia illustrated that Malaysian mothers viewed their child's autism symptoms and behavior problems as taking a toll on their well-being. They adopted coping strategies, like acceptance, proactive mindset, character growth, spirituality, parent support networks, and fostered well-being [14]. Similarly, caring for such child is overwhelming for Egyptian mothers also, due to the minimal services for children with disabilities. So, children with autism and their families face the likelihood of poor health, social care, mental health service, rehabilitation, lack of special education, and access to equal opportunities. In addition, individuals with autism are given little to no education because they either drop out of mainstream schools or parents cannot afford the scarce and expensive private schools. Meanwhile, parents receive a low level of support with regard to their children with ASD. Thus, mothers having children with autism spectrum are expected to experience greater burdens [15, 16]. Some mothers of Africa expressed their feelings towards themselves, others, and God, and recounted the use of forgiveness as a coping resource which contributed significantly to their well-being [17].

Autism is also common in Nepal. But studies related to autism are scant. The first article published on Autism reported that there is a lack of knowledge and awareness among parents and professionals regarding autism in Nepal [18]. Nepal is one of the low-income countries where the infrastructure for health services and education is limited. In Nepal, there are few organizations addressing and helping autistic children and their parents. So, it can be concluded that there is a great need for narrating and understanding the experience of mothers with autistic children to address the inadequacies in health, education, and social systems [19]. Hence, there is a great need to explore the lived experiences of mothers raising children with autism.

## 2. Materials and Methods

### 2.1. Research Design

Qualitative phenomenological research design was used to explore the experinece of mothers raising the children with autism. This phenomenology study design ensures an open attitude to the informant's life-world, while hermeneutics allows a participatory dialogue and inner subjectivity between the interviewer and informants [20]. It is done to understand the contexts within which mothers experience certain issues like autism. This design fits the exploratory nature of this study because it will allow for a detailed exploration of the mothers' experiences, as qualitative research acknowledges that there are multiple truths and that a person's interpretation of the experience is dependent on their social contex [21].

### 2.2. Research Setting and Population

This research study was conducted in participants' home setting located in Chitwan district, Nepal. Home setting was selected for the study in order to create an environment free from distractions with minimal interruptions where they will feel free to express their experience. The populations of the study were those mothers who have at least one clinically diagnosed autistic child aged less than fourteen years and attending care center such as Asha Bal Bikash Kendra or Autism Care Chitwan Society. Autism Care Society Chitwan is the branch of Autism Care Society Nepal that is run by passionate parents that care for children with autism. It is a nongovernmental, nonprofit making, nonpolitical NGO registered in District Administration Office with the vision of empowering people with Autism to protect and promote their rights and utilize their skills to have meaningful and effective participation in society. Asha Bal Bikas Kendra is a part of Human Development and Community Services established with the support of Norwegian Missionary, especially for children with physical and learning difficulties who are under the age of 16 years. It is established to develop basic life skills in order to improve the daily and acceptable level of social lives of all participating children with disability. It is situated in Milan Chowk, Chitwan. Both centers use therapies such as speech therapy, music therapy, vocational training, and day-to-day life activities to develop the physical and mental capabilities of children and grooming them to be independent adults.

### 2.3. Sampling

Qualitative research methods are often concerned with gathering an in-depth understanding of a phenomenon or are focused on a *meaning*, which is often centered on the *how* and *why* of a particular issue, process, situation, subculture, scene, or set of social interactions that occurred rather than many samples [22]. Phenomenologists tend to rely on very small samples, often 10 or fewer participants [23]. Nine mothers having at least one clinically diagnosed autistic child attending Autism Care Chitwan Society or *Asha Bal Bikas Kendra*, Bharatpur, Chitwan, Nepal, were taken as a sample of the study. Purposive sampling technique was used to select the sample. This purposive sampling technique was appropriate to select information-rich cases from which researcher obtained in-depth information needed to understand the experiences of mothers raising the children with autism in their day to day life. 

### 2.4. Inclusion Criteria

The inclusion criteria were defined as follows:

  Mothers having at least one clinically diagnosed autistic child
  Mothers willing to participate
  Mother having a child aged less than 14 years as the Labour Act (1992) puts the age limit of a child at 14 years

### 2.5. Exclusion Criteria

The exclusion criteria were defined as follows:

  Mothers not willing to participate voluntarily
  Mothers who have mental issues and who cannot communicate well

### 2.6. Data Collection Instrument

For the exploration of lived experiences, in-depth interview guidelines were developed by the researchers based on the review of related literature and consultation with the subject experts. In-depth interview guidelines consisted of eleven open-ended questions designed to elicit perceptions and experiences from nine mothers who had children with autism. For example “How do you perceive autism?” To investigate further, additional probing questions were used in the period of an interview as per need. Digital recorders and note books were used to record an interview and to take notes of special events that occur during the interview. The research instrument consists of two parts.

  Part I consists of questions related to sociodemographic information
  Part II consists of open-ended questions related to lived experiences of raising children with autism (attached in appendix)

### 2.7. Trustworthiness

To enhance the quality of data, an interview was conducted in simple Nepali language, and jargon was avoided as soon as possible. Verbal and nonverbal expressions were observed and noted during the interview by the researcher. The digital recorder was rechecked for its function and an interview was digitally recorded by the same recorder. Researcher preconceived ideas were bracketed during the data collection and analysis process. To ensure credibility or confidence in the truth of data, researchers conducted an in-depth interview and transcribed the obtained information along with field notes. Also, the researchers read the transcriptions several times to get a sense of the whole and reviewed field notes and digital recording. To maintain the dependability or stability of data, data were collected until it got saturated. Verbal and nonverbal expressions of the mothers were observed and recorded. On average, two to three interviews with each participant was conducted to ensure the same meaning was obtained from a different interview. Reconfirmation of the transcribed transcript verbatim was done by the researchers by going back to the respective participant to verify the meaning interpreted. Conformability was maintained by ensuring the meaning of relevancy during the process of transcription; results were discussed with a validated research advisor before the final interpretation. Transferability was achieved through the thick descriptions of mother's experiences along with their quotes and by using purposive sampling to maximize the information to be uncovered from few participants.

### 2.8. Data Collection Procedure

Proposal approval was taken from the Thesis Committee of School of Nursing, Chitwan Medical College, Bharatpur, Chitwan, Nepal. Ethical clearance (Reference Number 075/076–115) was taken from CMC-IRC. A request letter from CMC-School of Nursing was sent to *Asha Bal Bikas Kendra* and Autism Care Chitwan Society to get permission for data collection. After getting permission, researchers contacted mothers who met the study criteria individually to ensure their consent for participation and they were also explained about the objectives of the study. The date, time, and place for the interview were predetermined according to the convenience of mothers and they were approached by a reminder telephone call on the day of the interview.

Written informed consent, special permission for digital recording, and field notes were obtained from each mother before the interview. Participants were interviewed in their homes in a quiet place by using in-depth interview guidelines through the face-to-face interview method in simple Nepali language. In the beginning, general questions were posed to encourage the mothers to express their feeling and experiences of raising their children with autism. To ensure their clarity and in-depth information, further probing questions were asked in between. Each interview lasted for 45–60 minutes. Verbal and nonverbal expressions of the mothers were observed and noted during interviews. Each in-depth interview was recorded on a digital voice recorder to obtain a true account using the mother's own words. In addition, field notes were also maintained to capture other pertinent information during data collection. Two to three data collection sessions were carried for each mother and data collection was stopped after data got saturated.

### 2.9. Data Analysis Procedure

Data collection and analysis of data were done simultaneously. On the same day of the interview, a code number was assigned to the information sheet and pertinent information was recorded instead of personal identity. Then, the researcher stored the information sought safely. Two recordings were made to ensure that at least one copy would be available in the event of mechanical failure. Each interview was listened to repeatedly several times until the researcher herself became clear about it and transcribed verbatim at the end of the same day of the interview. Following transcription, each audio was listened to at least one further occasion by the researcher to verify the accuracy of the transcription. All interviews were first transcribed in the local Nepali language and then translated to English. After that, it was edited by an expert in both languages to ensure the quality of translation. The researchers immersed themselves in the data by reading transcripts multiple times and making memos about the key ideas that were mentioned by the mothers. All the unedited transcripts and comprehensive notes served as reference tools during the analysis of the investigation. After that, content analysis was done using Colaizzi's (1978) seven steps:

1. Rereading the transcriptions to obtain a general sense of the whole content
2. Extracting significant statements for each transcript that pertains to the phenomena under study
3. Formulation of meaning from significant statements
4. Making categories, themes, and a cluster of themes from formulated meanings
5. Integration of findings into an exhaustive description
6. Describing the fundamental structure of phenomena
7. Validation of findings from the participants to compare the researchers' descriptive results with their experiences; findings were presented in group interpretation in the report

## 3. Results

Table 1 shows that the mothers' age ranged from 23 to 36 years. All 9 mothers were from urban areas. Seven out of nine followed Hinduism. All mothers were educated. More than three-fourth were home-makers. Six out of nine mothers belonged to nuclear family. Six mothers received training on Autism and three were involved in informal classes about autism.

Table 2 represents demographic characteristics of autistic children. Among nine children, seven children were between ages 6 and 10 years. Six out of nine children were male and seven children were diagnosed at the age of three or four years. All nine children attend the care center. Likewise, six children were capable of some sorts of activity of daily living.

Table 3 shows the narrative description of a mother's experiences while raising children with autism. Five major themes and thirty-one minor themes emerged. They were perception about autism, recognition of problem modification in parenting style, impact on daily life, and coping strategies. Emerged themes and subthemes related to mothers' experiences of raising chidren with autism are shown with quotation from interviews. Pseudonym are given to participants instead of their real names to maintain the confidentiality of the information.

### 3.1. Perception about Autism

Mother's perception of autism was varied. They perceived autism as “mental retardation,” “life-long disorder,” “neurodevelopmental problem,” and “behavioral problem.” One participant aged 28 years, who was involved in an informal program of child care center, narrated her perception of mentally retarded condition as “*Autism is a mentally retarded condition where child behaves differently like he is slow, quarrels a lot unnecessarily, can't behave like a normal one, does not obey the given orders, does not listen to others and does something in his way. (P8)* Another participant aged 32 years, who took training on autism, explained her perception as “*It is neuro-developmental problem which appears since birth and the child fails to do a normal task, repeats the things continuously, unable to make eye contact and unable to respond when called.” (P3)*

### 3.2. Recognition of Problem

All participants reported poor developmental tasks and unusual behaviors such as avoiding speaking frankly, acting monotonously, disliking friends, staring continuously, repetition of things, avoiding eye contact, as early areas of concern for suspecting their child to be different from than that of normal. Problems in speech, communication, and socialization are the key features of atypical behavior observed by mothers among their children. Some mothers noticed the symptoms earlier whereas others recognized a little bit later. A 32-years-old participant narrated the experience of restricted and repetitive behavior as, “*By the time, he was two and half years old, he started showing different behaviors such as playing continuously with the same toy for a long time, did not care what was happening around. Also, he has a habit of saying the word ‘mummy' frequently . He is not close to other family members. He ignores if anyone tries to communicate with him. He gets scared of the Tempo sound. Observing all these things, I thought there should be something wrong with my child.” (P3)*

Similarly, another participant shared her experience of a child's problem in communication as *“Around 2 years of age, my child started to speak less. He speaks very limited words on his own, doesn't respond to us, and enjoys his solitary environment. He plays with a single thing for hours. He is okay with family members around him but dislikes any relatives who visited us at home. If any new person comes to the house, he cries till that person stays there. Once the person leaves, he becomes quiet again. All these circumstances made me recognize that my child had some problems.” (P6)*

In seeking to make the sense of the observed symptoms, most of the mothers (6/9) indicated that they used sources like the internet, newspapers as well as consulted neighbors having an autistic child. After suspecting the child's behavior, all mothers took their children to doctors for treatment. One 23-year-old participant who consulted her neighbors to make sense of her child's signs expressed her experiences “*At the age of 2 and half years, daughter started showing the different characteristics such as she was unable to complete the sentences, lacked patience, threw toys, etc. I consulted a neighbor who had also a child with a similar problem. He suggested to visit a doctor once. Later, my husband searched on internet and we found the symptoms matched with autism.” (P2*)

Simultaneously, all mothers reported that they visited temples and worshipped gods according to their own culture, hoping for a miracle. One mother aged 36 years who is religious and hoped that god would bring some changes in her son. She expressed her experiences as “*When I saw a problem in my child, I visited different temples, hoping for a miracle to happen. My child started speaking few words after I worshipped God. He might have spoken because of his developmental age but I thought it was due to my worship.” (P5)*

As per medical advice, three mothers disclosed that they took their child for speech therapy at Chitwan Medical College, Maharajgunj Hospital, and only one mother took her child for vocational training. Those who took their child for speech therapy described that they were not satisfied with therapy as it did not produce an effective outcome on their child's communication.One mother expressed her feeling as “*As per the doctor's advice, I took my child for speech therapy for fifteen days in the health care center but I did not see any sorts of improvement in my child. So, I discontinued the therapy .” (P1)*

### 3.3. Modification in Parenting Style

In order to modify the parenting style, the mother followed the advice given by doctors (physician, pediatrician, or neuro-medicine doctors). Six participants disclosed that they took their children for Autism training to teach them about behavior modification activities, physical activities, play therapy, and some sort of regular classes (matching, sorting, recognizing body parts, weather, colors, animals, fruits, etc.). In addition, participants disclosed that training produced some good outcomes for their children. One participant said, *“Earlier, he used to throw things frequently, get scared with tempo sound and very difficult to convince him for cutting his hair. But after training, these sorts of habits slowly faded away.” (P3)*.

Similarly another mother expressed her feeling as “*Previously, he was disobedient. He used to beat everyone he saw, pulled clothes, used to urinate and excrete anywhere. But after getting training on activities like washing hands, brushing, toileting, spending time with friends, recognizing objects and body parts, matching, and color identification, he is able to sit on his own, ask for help with visual aids and started to obey orders which led me to believe that there is some improvement.” (P4)*

Majority of mothers (6/9) took training on handling and caring techniques of child, speech, and vocational training which aids them to bring some changes in their parenting style, especially on their behavior, daily activities (like eating, sitting, washing face, toileting, wearing dress, etc.). In addition, they disclosed that it helps them to understand more about their child's strength, emerging skills, and weakness. Use of visual aids for signals, encouragement, and rewards, acting as guard, keeping patience, teaching with repetitions, and keeping child busy with toys, mobile, and T.V. were some of the ways that mothers adopted in caring their children. One mother shared her experience as “*Earlier, I was unaware about caring and handling techniques of child. I used to get angry, shout and beat him when I saw his irritating behaviours like playing with soap and surf, tearing pillow cover and bed sheets, beating his elder sister, eating whatever he found. I used to lock the door and cry alone. But after training, I came to know that I should not beat him, rather I should control myself with positive thoughts. I realized these children didn't know what is wrong and what is right so, it's duty of mother to teach them without anger.” (P4)*

However, some participants who only took informal educational classes conducted in child's care centers were also satisfied with the classes provided from there. One participant aged 32 years who took informal class narrated as *“Although, I didn't take training on autism, I used to get invited in the informal program. So, I got chance to learn about handling and caring techniques, ways of behaving, treating and teaching child with autism.” (P7)*

### 3.4. Experiences in Daily Life

#### 3.4.1. Physical Problems

It is not easy for a parent to look after the child with autism, as their parenthood journey is different from what they hoped for. Participants in the study faced physical toil in their daily life due to continuous monitoring of their children. One 32-year-old participant who faced physical toil reflected her experiences as *“Really, it's very difficult to look after my child because she is not able to perform daily activities. I devote every moment to her care. So, I wake up early in the morning to finish household tasks before she wakes up. It is very difficult to have sound sleep at night which makes things even harder during the day.”(P9)*

#### 3.4.2. Emotional Problems

The emotional burden was identified among all mothers when they were asked about the overall impact of raising a child with autism. Some of the mothers in the study became tearful during interviews when describing their feelings. They reported overwhelming emotional reactions to their child's diagnosis as they were unable to accept the child's diagnosis initially. Mothers further narrated that the period before diagnosis was more difficult for them due to limited knowledge for the management of the atypical behaviors of their children. The daily struggles related to the child's caregiving, and thoughts about the child's future made them anxious. Moreover, accepting the diagnosis of autistic children was more challenging for every mother. One mother narrated her feelings as “* Initially, I refused to accept my child as autistic. I thought it was something else, causing my child to behave differently. So, rather than accepting the fact, I started searching other reasons for my child's behavior. I insisted the doctor to undergo every investigation to seek out the true reason behind my child's condition. But later, every report came to be normal.” (P2)*

Some mothers experienced a sense of relief to some extent after the diagnosis of their child whereas other felt a loss of sense when they first learned that their child will not be able to experience life as other children do. One mother with such emotions narrated her feeling as “*Once my son was diagnosed to have autism, I was very much depressed . Everytime I attended any social event, people used to ask me about my child's condition which made me more depressed.” (P1)*

In addition, anger was also an acute reaction expressed by mothers when they were not getting help from family members and when child's behavior was hard to handle. Some mothers disclosed that sometimes they became irritated when their child interrupted them in household activities. One 36-year-old participant expressed her sense of displeasure as, “*I have another child also. So, I used to get very angry when these two kids quarrelled with each other. I have even punished them. In addition, it's very hard for me to get involved in household tasks because he always accompanies me and interferes in my daily activities.” (P8)*

Parenting the children is stressful, and taking care of a child with special needs is often more so. Negative emotions are normal. Indeed, the transition to adolescence generated anxiety for all mothers regardless of the child's age. Moreover, uncertainty about the child's education, career, living conditions, and adaptation to adulthood contributed to an intense sense of worry. The essence of her future-oriented fears was reported by mother as “*My child is not able to speak well. I am more worried about her future as she is girl child. She is growing day by day and physical changes are obvious. Thought of what will she do and how will she express herself during her menstruation period bothers me. I may not be always by her side, one day I will get old and die and how will she live, how will she perform her activities.” (P9)*

#### 3.4.3. Social Problems

Mothers with autistic children not only experienced physical and emotional problems. They also suffer from social problems such as social ignorance (2/2), social blame (5/5), and social isolation (7/9) due to atypical behavior of the child, fear of judgment, and stigmatization by others. One 32-year-old mother shared her experience of social blame as “*People in the community blamed me that the condition of the child was due to my smartness, unnecessary pampering, and over-confidence. They even told that child should be scolded and beaten to make him obedient. Likewise, other people used to say it was due to dietary negligence during pregnancy.” (P6)*

Mothers reported a significant decrease in the quantity and quality of their social ties and relationships due to increased childcare responsibilities and potential behavioral outbursts of child in public. A mother who faced social isolation shared her experiences as “* I am very much affected by my child's condition. I don't have my own life at all as I have to look after him every moment. I couldn't attend any parties leaving him alone at home. If I took him, he would behave improperly at those places like throwing food, beating people, pulling clothes. I have to be beside him every time like a guard.” (P4)*

The majority of mothers described that having a child with autism not only affects the mother's life but also has an impact on the sibling's daily activities and emotions. In addition, mothers reported the feeling of guilt related to neglect, inattention, and inadequate parenting of their unaffected child. One mother expressed her feeling as “*My elder son is autistic. The younger one is too small to understand his brother's condition. Autistic son also doesn't understand that he is the elder one so, he should love and care for his brother. Both quarrel for small things like play materials, mobile phones. So, I should spend much of the time pampering him. As a result, I am unable to give much of the time to the younger one which made me feel bad. During day time also, I need to attend care center along with elder one leaving younger son at home.” (P6)*

#### 3.4.4. Economic Problems

The majority of mothers faced economic problems due to limited familial income as only one person is earning in the family and they had to spend a huge chunk of earnings on child's treatment and therapies. Mothers also felt an impact on their careers due to their child's condition. Most mothers stated that it was impossible to join a job along with the care of a child. This led some of the mothers to feel that they were unable to help their child financially for specialized intervention. One mother narrated her problem as “*I have made my education license thinking that I could join job somewhere but I am unable to join yet because of her such condition. So, only one person is earning for the family and we are having difficulty in managing household expenditure as well as child's treatment. So, I am unable to afford extra therapies for her. Financial hardship even sometimes leads to disputes with my husband.” (P7)*

### 3.5. Coping Strategies

All mothers reported that they tried to cope with their situation by using different coping measures such as respite care, problem-focused strategies (employment, training on autism, attending program related to autism, initiation of care center, social support), positive coping (remaining hopeful for future, setting a goal, use of treatment services), and religious coping.

#### 3.5.1. Respite Care

Respite care is one of the coping methods used by all mothers, i.e., they had admitted their child in care centers and schools so that they would feel relief from tension and get free time for a certain period. They also believed that their child gets better assistance and learns something new from the center. One mother reported her coping measure of respite care as “*I admitted my child in daycare center so; I am free whole day and could engage myself in household tasks.” (P9)*

#### 3.5.2. Religious Coping

One of the most important strategies mentioned by the majority of mothers was spiritual coping, i.e. strong belief in god hoping for the betterment of the child. One mother aged 34 years accepted her child's condition and said “*I believed that autistic children are the gift from god. The outcome of this gift depends upon how well we decorated them. If we decorate them in a good manner, we will get a good outcome otherwise the outcome will be poor.” (P4)*

#### 3.5.3. Positive Coping

It has been seen that mothers surrounded themselves with positive thoughts to cope up with the problems they faced. As a part of positive coping, mothers positively reframe their thoughts by keeping some hope and determination that they would do something good for, till they are alive. One mother expressed her feeling as “*What I think is, we should never indulge ourself on same thing, instead we should move on keeping our past aside. We should try to forget our past considering it as bad dream. I surround myself with positive feelings that I can do something good for my child, which is only the goal of my life. The more we keep thinking about the problem, the more we cannot move on. So, we should think positive and move on as if nothing has happened.” (P2)*

#### 3.5.4. Problem-Focused Strategies

Problem-focused strategies focused on managing and altering the stressors of rearing their autistic children, which includes social support and action to deal with the problem. All mothers reported that they took social support from family members, relatives, and biological parents as needed. In addition, one mother reported that she had initiated an “Autism Care center” to deal with her problem and help other similar mothers and children. A 32-year-old mother narrated her feeling as *“With the aim of helping these kinds of children and their mothers, I collaborated with mothers of autistic children to initiate a care center. I stay whole day in the care center with my child to teach him various activities of daily living. Occasionally, I attend programs related to autism, conducted by the municipality and some other governmental organizations also.” (P6)*

## 4. Discussion

Here, the discussion deals with all comprehensive approaches of findings, conclusion, and recommendations. Findings are discussed with the established facts and published studies are related to lived experiences of mothers raising children with autism. The objectives of the study were to explore the lived experiences of mothers raising children with autism in their day-to-day life. Nine mother's experiences with autistic children were explored through in-depth interviews and their experiences were interpreted reflecting their problems and responses to overcome the situations.

### 4.1. Perception about Autism

The first major theme emerged from this study involved the perception of mothers on autism. There was a varied experience of mothers' perception on autism. Each mother has her own way of understanding the problem, i.e., behavioral problem, developmental problem, and mental retardation, which require life-long care, assistance, and attention. The current findings support previous studies that found overwhelming perception of mothers on autism as a developmental disability and believed that it is caused by several factors including developmental delay, medical disorder, genetic disorder, and neurodevelopmental problems that begin before birth or infancy [24].

### 4.2. Recognition of Problem

In the present study, mothers were the one who first recognized the child's problem through the child's behavior based on the following symptoms such as isolation from friends and family, enjoying own solitary environment, playing alone continuously for hours with the same toy, ignoring other people, unable to make eye contact and acting monotonously which led them to suspect their child's abnormal condition. It displays similar findings to the study conducted in New York which reported that manifestations of autism vary greatly depending upon the developmental and chronological age of the child which may include several behavioral symptoms such as hyperactivity, impulsivity, and aggression including self-injurious behavioral and temper tantrums [25].

With regard to the help-seeking behaviors mothers engaged in after first becoming concerned with their child's atypical behaviors and developmental delays, the majority of mothers had first brought their concerns to their child's pediatrician. Others simultaneously began to research the child's signs on the Internet or by reading magazines, comparing them with siblings and neighbors' child. Similar finding is shown in the study conducted in the UK and USA where mothers were confused by their children's behavioral difficulties and developmental delay. So, they relied on parenting books, the Internet, as well as consulted other women having autistic children to explore more about the problem as well as about their experiences in rearing and caring for their children. These mothers were most likely to contact the child's physician first who supported them and facilitated the diagnostic process [26, 27].

Of the mothers who sought the counsel of pediatrician, they reported that pediatricians took their concern seriously and provided them a referral to autism clinic for therapies and training. Seven mothers who trained their children reported that they saw significant changes in their child's behavior and developmental tasks after the training. Similar findings are shown in the study conducted in the United States and Canada where targeted therapies like speech/language, occupational therapy, and vocational training help to increase communication skills, improve independence in activities of daily living, and improve social and behavioral adjustment. Furthermore, parents' training interventions help to foster children's development by enhancing parent-child interactions. It also elaborated that those parents who received parent training interventions for their young child with Autism were able to learn about it and implement strategies to foster their child's development and were very satisfied with the training [28].

### 4.3. Experiences in Daily Life

#### 4.3.1. Physical and Emotional Problems

Rearing and caring for differently abled children is a great challenge for mothers. They suffer the most as they have to devote their every moment caring for them which led to physical problems such as exhaustion, sleep disturbance, and loss of appetite. Besides physical problems, mothers faced emotional problems like denial during early diagnostic phase, anxiety regarding child's future, and being upset due to the child's disability. Mothers were worried that they did not really know what the future would bring for their child. This could be due to the fact that there is no established organization to provide support for children. Many mothers expressed the hope that their child could have a “normal” life or could acquire some skill to live an independent life. Majority of mothers had already started to think about the life skills (skills needed for activity of daily living) their child would need to be independent. This is similar to findings of a study conducted in Turkey which showed that mothers experienced rejection as the initial reaction after the diagnosis of a child [29]. Likewise, a study conducted in Africa found that initially the parents denied the diagnosis, doubted the diagnosis due to a lack of understanding of the disorder, or did not believe that such a thing would ever occur to their child [30]. Similarly, the study conducted in Greece reported that mothers were the primary caregivers and perceived their major role as providing and coordinating care for the child in combination with other daily activities, including housekeeping and caring for the other family members' needs. These numerous caregiving duties negatively impacted maternal mood and well-being, and created feelings of helplessness and powerlessness that increased pessimism regarding the future of their affected child [31].

#### 4.3.2. Social Problems

Mothers' lives were affected socially as well as economically by having autistic children. A sense of feeling isolated was common among these mothers as they were unable to participate in social activities because most of their time was devoted to the care of their autistic children, and they believed that taking them to religious ceremonies and other formal functions would be deemed inappropriate due to the antisocial behavior that are common traits of autism. Moreover, mothers described that they were scolded by family and others and blamed that their negligence, over-smartness, and unnecessary pampering were the causes for the child's disability. All these problems not only affect parents' lives but also have negative impacts on siblings, which causes significant change in the entire family system, including an increase in problems with sibling and neighbor's relationships. This is consistent with findings of the study conducted in the USA which showed that mothers get socially deprived as they could no longer relate to their old friends due to lack of time to spend socially, an inability to bring children to social gatherings due to behavioral concerns and fear of judgment or stigmatization [32]. Moreover, the people in the community were more sensitive to the behaviors of autistic children and blamed mother's behaviors and personalities as the potential causes of autism [33].

#### 4.3.3. Economic Problem

Children diagnosed with autism need continuous care and services, regular monitoring, and treatment therapies in their daily life. As a result, either of parents has to give up their job which can be financially stressful as it leaves only one parent to support the entire family. Economical problem was also seen among the mothers included in the study. Mothers were unable to continue their job due to the child's disability. Besides, they had to spent huge chunk of money on treatment, therapies, and play materials which were costly. The idea that mothers are economically distressed echoes previous qualitative work, which indicated that 24% of families of autistic children need to reduce their work hours or completely stop working because of continuous caring and supervision of their child [34]. In addition, families of children with ASD incurred higher health-related costs compared with families of children with other special health care needs [35]. Similarly, a study conducted in Greece reported that mothers are forced to make significant adjustments in lifestyle because of various factors, such as poor insurance coverage for needed services and higher costs for intense interventions (i.e., speech and occupational therapy, psychotherapy) [31].

#### 4.3.4. Coping Strategies

Considering all of these sorrowful experiences through an ecological lens, a number of systems are impacting the mothers' experiences in positive ways. Mothers reported that coping strategies, including autism care society, assistance from their biological and in-laws families and friends, and the support of their faith or religious community, as having a positive impact on their raising of children. The strategies used by mothers to adjust with stressors they faced in their day-to-day life are divided into four aspects.

The first one is respite care. All mothers adopted respite care as they had admitted their children to the care center. This is supported by the findings of the study conducted in Sydney, which showed that respite care can help reduce stress in families as well as provide them with the chance to have a break from caring and doing things that they cannot do while the child with ASD is at home [36].

Secondly, mothers adopted problem-focused strategies (sought medical help and social support), whereas one mother initiated a care center to help autistic children. These findings are consistent with findings of the study conducted in England, which showed the use of problem-focused strategies (e.g., treatments/interventions for the child, social support) by mothers of children with ASDs [37].

Thirdly, all mothers relied on religious coping, hoping for a miracle in their child's condition. They visited various temples and performed rituals. This is similar to the findings of a study conducted in India which found that intervening factors like optimism, faith in gods, and religious support help to alleviate the degree of stress, generate psychic energy to cope with the physical, emotional, and financial aspects of caregiving among mothers having ASDs children [38].

Lastly, positive coping is seen as another strategy used by mothers to minimize the stress while rearing children with autism. These findings are similar to the findings of the study conducted in India, which showed that positive perceptions play a central role in the coping process which assists them in dealing with the stressful events caused by a child's disability. Side by side, it showed that positive perceptions lead to a better quality of life for those families and provide them good scope for maximizing the child's potential [39].

## 5. Conclusions

Based on major findings, it is concluded that the journey of the mother while rearing and caring for children is challenging. Each mother has a unique experience with their child's disability. They experience physical exhaustion, sleep disturbance, and loss of appetite as well as they become emotionally disturbed during the early diagnostic phase and suffer from fear of judgment in public events, anger due to child's annoying behavior, and become worried about their child's future. Costly medical and therapeutic intervention poses huge financial problems for them. To adjust in daily life, mothers tend to adopt various coping strategies.

### 5.1. Implications

The finding of the study would be helpful to educate nurses/other health care providers about the shared experiences of families with autistic children that will help to provide need-based care to individuals with autism and their families. It might also be helpful for policymakers and health care planners to plan and intervene in the program to address the need of those mothers with autistic children. This study would also serve as baseline information for future researchers. The findings related to coping strategies used by autistic mothers would be helpful to other similar parents for adjustment in their daily life.

### 5.2. Limitations

- The nature and amount of information the mothers offered depend upon their interactions with the interviewers, the circumstances surrounding the interview, and their motivation for participating in the study
- Due to the sensitive nature of the study, respondents might have not shared all of their opinions, and their responses might be different

### 5.3. Recommendation

Mothers reported many problems such as physical, emotional, social, and economic problems in their day-to-day life while rearing children with autism. So, this finding suggests close attention and guidance to the mothers by health care providers during their child's treatment.

Some additional research on autistic children is required to cover the larger geographical settings to address the limitations of this study.

Public awareness programs through media related to autistic children should be launched to make everyone familiar with this condition.

It is recommended to conduct a similar type of study employing a mixed-method design incorporating both qualitative and quantitative methodology to allow for generalization by covering many more areas to make findings more valid in a different setting.

Further research study is recommended to explore the experiences of mothers whose autistic children are in the community using snowball sampling.

## Figures and Tables

**Table 1:** Sociodemographic information of the mothers.

SN	Name of participant	Age (in years)	Residence	Religion	Educational status	Occupational status	Socioeconomic status	Types of family	Receive training
1.	Tinu	34	Urban	Hinduism	Bachelor	Home maker	Adequate	Joint	Yes
2.	Mamita	23	Urban	Hinduism	Basic up to 8 class	Home maker	Inadequate	Nuclear	Yes
3.	Parita	32	Urban	Hinduism	Bachelor	Home maker	Adequate	Nuclear	Yes
4.	Sashi	34	Urban	Hinduism	Secondary level	Home maker	Adequate	Nuclear	Yes
5.	Tina	36	Urban	Buddhism	Basic up to 8 class	Job holder	Adequate	Nuclear	Yes
6.	Devi	32	Urban	Hinduism	Bachelor	Job holder	Adequate	Nuclear	Yes
7.	Amrita	32	Urban	Hinduism	Secondary level	Home maker	Inadequate	Nuclear	No
8.	Salina	28	Urban	Hinduism	Bachelor	Home maker	Adequate	Joint	No
9.	Kabita	32	Urban	Islam	General literate	Home maker	Inadequate	Joint	No

Mean age ± SD = 31.44 ± 3.62, range = 23–36 years.

**Table 2:** Characteristics of autistic children (*n* = 9).

Variables	Frequency
*Age group (years)*	
6–10	7
11–15	2

*Sex*	
Male	6
Female	3

*Age at the time of diagnosis of child*	
3 years	3
4 years	4
5 years	1
12 years	1

*Involvement of child*	
Care center	9

*Capable of self-care*	
Yes	6
No	3

Mean age ± SD = 8.55 ± 2.05, range = 6–12 years.

**Table 3:** Emerged theme on lived experiences of mothers with autistic children.

Major theme	Minor themes
Perception about autism	(i) Mental retardation
	(ii) Behavioral problem
	(iii) Lifelong disorder
	(iv) Neurodevelopmental problem

Recognition of problem	(i) Problem in socialization
	(ii) Problem in communication
	(iii) Restricted or repetitive behavior
	(iv) Poor developmental tasks
	(v) Sources of information

Modification in parenting style	(i) Changes in activity of daily living
	(ii) Capable of developmental tasks
	(iii) Positive reinforcement
	(iv) Uses of different resources
	(v) Safety precautions
	(vi) Perceiving child's compatibility

Impact on day-to-day life	Physical aspects
	(i) Physical toil
	Emotional aspects
	(i) Denial
	(ii) Worry
	(iii) Sad
	(iv) Anger
	(v) Anxious
	(vi) Relief
	Social aspects
	(i) Isolation
	(ii) Ignorance
	(iii) Social blame
	Economic aspects
	(i) Economic hardship
	(ii) Loss of job opportunity

Coping measures	(i) Respite care
	(ii) Problem-focused strategies
	(iii) Spiritual coping
	(iv) Positive coping

## Data Availability

The audio clips are confidential and accessible to researchers only.

## Appendix

### A. DSM-5 Diagnostic Criteria for Autism Spectrum Disorder

(A) Persistent deficits in social communication and social interaction across multiple contexts, as manifested by the following, currently or by history:
  1. Deficits in social-emotional reciprocity
  2. Deficits in nonverbal communicative behaviors used for social interaction
  3. Deficits in developing, maintaining, and understanding relationships
(B) Restricted, repetitive patterns of behavior, interests, or activities, as manifested by at least two of the following, currently or by history:
  1. Stereotyped or repetitive motor movements, use of objects, or speech
  2. Insistence on sameness, inflexible adherence to routines, or ritualized patterns of verbal or nonverbal behavior
  3. Highly restricted, fixated interests that is abnormal in intensity or focus
  4. Hyper- or hyporeactivity to sensory input or unusual interest in sensory aspects of the environment
(C) Symptoms must be present in the early developmental period (may not become fully manifest until social demands exceed limited capacities, or may be masked by learned strategies in later life)
(D) Symptoms cause clinically significant impairment in social, occupational, or other important areas of current functioning
(E) These disturbances are not better explained by intellectual disability (intellectual developmental disorder) or global developmental delay

### B. Semistructured Interview Guidelines

(1) How do you perceive autism?
(2) When did you recognize that your child has some problem?
(3) What did you do when you realized that your child had problem?
(4) What were your experiences at the time of diagnosis and treatment?
(5) What measures are being used in the treatment of your child?
(6) What improvement have you seen in your child after the treatment?
(7) What changes have you adopted in your parenting styles after diagnosis and treatment?
(8) Could you please tell me about the negative and positive aspects you have faced while raising your autistic child?
    Probing Questions
  (i) Physical aspects: physical burden faced by mothers in their daily life
  (ii) Psychological aspects: anxiety, sad, isolation, self-blame, depression
  (iii) Social aspects: relationship with family members, other siblings and neighbors, job and carrier
  (iv) Financial aspects: cost expended on child's treatment, play material, study, etc.
(9) Could you please share me how you have adjusted in your daily life while raising your autistic child?
    Probing Questions
  (i) Behavioral activity of child
  (ii) Caring in activity of daily living
  (iii) Treatment cost
(10) Would you please specify the coping measures that you have employed to relieve your problems while dealing with your autistic child?
(11) Could you please explain me about the support system you have gained while raising the autistic child?
  - Family, community
  - Self-help group
  - Organization
(12) Would you like to share anything else with me that we have forgotten to discuss till now?
